# Next-Generation Sequencing–Based Genomic Profiling of Children with Acute Myeloid Leukemia

**DOI:** 10.1016/j.jmoldx.2023.04.004

**Published:** 2023-08

**Authors:** Szilvia Krizsán, Borbála Péterffy, Bálint Egyed, Tibor Nagy, Endre Sebestyén, Lajos László Hegyi, Zsuzsanna Jakab, Dániel J. Erdélyi, Judit Müller, György Péter, Krisztina Csanádi, Krisztián Kállay, Gergely Kriván, Gábor Barna, Gábor Bedics, Irén Haltrich, Gábor Ottóffy, Katalin Csernus, Ágnes Vojcek, Lilla Györgyi Tiszlavicz, Krisztina Mita Gábor, Ágnes Kelemen, Péter Hauser, Zsuzsanna Gaál, István Szegedi, Anikó Ujfalusi, Béla Kajtár, Csongor Kiss, András Matolcsy, Botond Tímár, Gábor Kovács, Donát Alpár, Csaba Bödör

**Affiliations:** ∗ HCEMM-SE Molecular Oncohematology Research Group, Department of Pathology and Experimental Cancer Research, Semmelweis University, Budapest, Hungary; † Department of Pediatrics, Semmelweis University, Budapest, Hungary; ‡ Department of Biochemistry and Molecular Biology, Faculty of Medicine, University of Debrecen, Debrecen, Hungary; ‡‡ Department of Pediatric Hematology and Oncology, Institute of Pediatrics, University of Debrecen, Debrecen, Hungary; §§ Department of Laboratory Medicine, University of Debrecen, Debrecen, Hungary; § Hemato-Oncology Unit, Heim Pal Children’s Hospital, Budapest, Hungary; ¶ Division of Pediatric Hematology and Stem Cell Transplantation, Central Hospital of Southern Pest, National Institute of Hematology and Infectious Diseases, Budapest, Hungary; ‖ Department of Pediatrics, University of Pécs Clinical Centre, Pécs, Hungary; ∗∗ Department of Pediatrics and Pediatric Health Care Center, University of Szeged, Szeged, Hungary; †† Hemato-Oncology and Stem Cell Transplantation Unit, Velkey László Child’s Health Center, Borsod-Abaúj-Zemplén County Central Hospital and University Teaching Hospital, Miskolc, Hungary; ¶¶ Department of Pathology, University of Pécs Clinical Centre, Pécs, Hungary; ‖‖ Division of Pathology, Department of Laboratory Medicine, Karolinska Institutet, Karolinska University Hospital, Stockholm, Sweden

## Abstract

Pediatric acute myeloid leukemia (AML) represents a major cause of childhood leukemic mortality, with only a limited number of studies investigating the molecular landscape of the disease. Here, we present an integrative analysis of cytogenetic and molecular profiles of 75 patients with pediatric AML from a multicentric, real-world patient cohort treated according to AML Berlin-Frankfurt-Münster protocols. Targeted next-generation sequencing of 54 genes revealed 17 genes that were recurrently mutated in >5% of patients. Considerable differences were observed in the mutational profiles compared with previous studies, as *BCORL1*, *CUX1*, *KDM6A*, *PHF6*, and *STAG2* mutations were detected at a higher frequency than previously reported, whereas *KIT*, *NRAS*, and *KRAS* were less frequently mutated. Our study identified novel recurrent mutations at diagnosis in the *BCORL1* gene in 9% of the patients. Tumor suppressor gene (*PHF6*, *TP53*, and *WT1*) mutations were found to be associated with induction failure and shorter event-free survival, suggesting important roles of these alterations in resistance to therapy and disease progression. Comparison of the mutational landscape at diagnosis and relapse revealed an enrichment of mutations in tumor suppressor genes (16.2% versus 44.4%) and transcription factors (35.1% versus 55.6%) at relapse. Our findings shed further light on the heterogeneity of pediatric AML and identify previously unappreciated alterations that may lead to improved molecular characterization and risk stratification of pediatric AML.

Although pediatric acute myeloid leukemia (AML) constitutes only 20% of cases, it accounts for >40% of childhood leukemic mortality.^1^ AML represents a heterogeneous malignant disease of myeloid progenitor cells characterized by the acquisition of recurrent genetic alterations. With the optimization of intensive chemotherapy regimens, treatment of pediatric AML has improved over the past decades, with current survival rates reaching 70% in developed countries. However, 30% of patients encounter relapse that still represents a major therapeutic challenge.^2^, ^3^, ^4^, ^5^, ^6^, ^7^, ^8^ With the advent of high-throughput technologies, identification of molecular alterations has revealed considerable molecular heterogeneity, both between and within individual patients, and expanded our knowledge of mechanisms underlying leukemogenesis, clonal expansion, and treatment resistance of leukemic cells.^9^, ^10^, ^11^ The widespread utilization of next-generation sequencing (NGS) led to the comprehensive characterization of patients with AML and to the discovery of novel, clinically relevant recurrent molecular aberrations, some of which represent important prognostic and predictive biomarkers.^6^^,^^9^^,^^11^

Most reported studies investigating the molecular genetics of AML have focused on adult patients, with limited applicability to pediatric AML, as genetic profiles of adult and pediatric patients are substantially different.^12^^,^^13^ Several studies showed that pediatric and adult AML cases both have relatively low somatic mutation burden compared with other malignancies; however, genetic alterations most commonly occurring in adults are less frequently observed in pediatric patients with AML.^13^, ^14^, ^15^, ^16^, ^17^ Overall, the genetic landscape of pediatric AML is relatively undercharacterized due to the limited number of studies investigating the genetic background of childhood AML.^13^^,^^14^^,^^18^, ^19^, ^20^, ^21^, ^22^, ^23^, ^24^

In the current study, we performed mutational profiling of 54 target genes in a real-world cohort of patients with pediatric AML using a panel-based targeted NGS approach combined with cytogenetic data obtained via standard karyotyping. The aim was to genetically characterize these patients and to investigate the clinical impact of the identified variants. Therapy-induced clonal evolution of leukemic cell populations was analyzed by using samples collected sequentially from the same patients at diagnosis and relapse. We describe the integrative cytogenetic and molecular profile of this pediatric AML cohort and show the widespread applicability of clinically relevant, NGS-based mutation profiling in the genetic characterization of pediatric AML.

## Materials and Methods

### Patients and Samples

Seventy-five patients with pediatric AML were included in this study, with a female:male ratio of 1:1.2. The median age at diagnosis was 9.0 years (range, 0 to 17 years), and the median white blood cell count was 8.9 × 10^9^/L (range, 0.51 to 348 × 10^9^/L). Basic clinical and cytogenetic features of the patient cohort are summarized in Supplemental Table S1. Diagnostic bone marrow (*n* = 70) or peripheral blood (*n* = 2) samples from 72 children diagnosed with AML, as well as skin (*n* = 1) or lymph node (*n* = 2) samples from 3 children diagnosed with extramedullary AML, were analyzed. Diagnoses were established based on morphologic, immunophenotypical, and genotypical criteria at the Department of Pathology and Experimental Cancer Research, Semmelweis University, in the Department of Pathology, University of Pécs, or in the Department of Pathology, University of Debrecen, between 2003 and 2021 according to the classification system of the World Health Organization.^25^ Patients were risk stratified and treated according to Berlin-Frankfurt-Münster (BFM) protocols, including AML-BFM 98, AML-BFM 2004, AML-BFM 2012, and AML-BFM 2019 (Supplemental Table S2). In addition to the diagnostic samples of 74 patients, nine samples drawn at the time of first relapse and three samples drawn at second relapse were investigated.

The median follow-up time was 23.8 months (range, 0.2 to 205.0 months). Additional clinical characteristics of patients are summarized in Supplemental Table S2. DNA was extracted from the corresponding specimens (bone marrow, *n* = 79; peripheral blood, *n* = 3; skin, *n* = 1; lymph node, *n* = 2; and cerebrospinal fluid, *n* = 1) using the High Pure PCR Template Preparation Kit (Roche Life Science, Indianapolis, IN). Extracted DNA was quantified by using the Qubit dsDNA HS assay kit and Qubit 4.0 Fluorometer (Thermo Fisher Scientific, Waltham, MA).

#### Ethics Statement

Written informed consent was obtained from the patients’ parents or guardians. The study was approved by the Medical Council Research of Hungary (IV/51-1/2022/EKU), and it was conducted in accordance with the Declaration of Helsinki.

### Cytogenetic Analysis

Cytogenetic aberrations were determined by conventional G-banding and fluorescence *in situ* hybridization to detect abnormalities related to AML. Probes for *RUNX1::RUNX1T1*/t(8;21)(q22;q22) (ZytoVision, Bremerhaven, Germany), *CBFB::MYH11*/inv(16)(p13.1q22)/t(16;16) (p13.1;q22) (Abbott Molecular, Des Plaines, IL); *PML::RARA*/t(15;17)(q22;q12) (ZytoVision), and *KMT2A*/11q23 rearrangements (ZytoVision) were used for fluorescence *in situ* hybridization analysis. A complex karyotype was defined as three or more chromosomal aberrations in the absence of the recurrent AML genetic aberrations defined by using the World Health Organization classification, including t(8;21)(q22;q22), inv(16)(p13.1q22)/t(16;16)(p13.1;q22), t(15;17)(q22;q12), t(6;9)(p23;q34.1), *KMT2A*/11q23 rearrangement, or t(9;22)(q34;q11.2). Patients with core-binding factor (CBF)-AML characterized by inv(16)(p13;1q22), t(16;16)(p13;q22), t(8;21)(q22;q22), and t(15;17) (q24;q21) were categorized in the favorable risk group; complex karyotype, monosomy 7, t(6;9)(p23;q34.1), inv(3) (q21q26), t(4;11)(q21;q23), t(6;11)(q27;23), and t(10;11) (p12;q23) were considered as adverse prognostic markers. Patients with normal karyotype and cytogenetic abnormalities not included in the aforementioned subgroups were classified into the intermediate-risk group.

### Assessment of *FLT3*-Internal Tandem Duplication and *CEBPA* Mutational Status

Mutation analysis of *FLT3*-internal tandem duplication (ITD) was performed from genomic DNA using primers adapted from Kottaridis et al^26^ (11F: 5′-FAM-GCAATTTAGGTATGAAAGCCAGC-3′; 12R: 5′-CTTTCAGCATTTTGACGGCAACC-3′). Fluorescently labeled PCR products were analyzed by using a capillary electrophoresis 3500 Genetic Analyzer (Thermo Fisher Scientific) and GeneMapper software 5 (Thermo Fisher Scientific). The mutant *FLT3*-ITD allelic burden was calculated as the ratio of area under the mutant versus wild-type *FLT3* peak. For the assessment of the mutation status of *CEBPA*, the entire coding sequence was amplified by using three overlapping PCR fragments with previously described primer pairs [forward (F)1: 5′-TCGCCATGCCGGGAGAACTCTAAC-3′, reverse (R)1: 5′-AGCTGCTTGGCTTCATCCTCCT-3′, F2: 5′-GCTGGTGATCAAGCAGGAGC-3′, R2: 5′-CCGCCACTCGCGCGGAGGTCG-3′, F3: 5′-GGCAGCGCGCTCAAGGGGCTG-3′, and R3: 5′-CACGGCTCGGGCAAGCCTCGAGAT-3′] and analyzed by using bidirectional Sanger sequencing.^27^

### Targeted NGS

Targeted NGS was performed by using the TruSight Myeloid Sequencing Panel (Illumina, San Diego, CA) covering 54 leukemia-associated genes (Supplemental Table S3). Individual libraries were prepared from 50 ng of genomic DNA according to the manufacturer’s recommendations. DNA extracted from peripheral blood mononuclear cells of 15 healthy volunteers with normal complete blood profiles were used as negative control subjects. After quality control and equimolar pooling, libraries were sequenced on a NextSeq 550 platform (Illumina) using v2.5 chemistry with 150 bp paired-end configuration.

### Bioinformatics Analysis

Raw sequencing data generated from TruSight Myeloid libraries were analyzed by using the TruSeq Amplicon app in BaseSpace Sequence Hub (Illumina). After demultiplexing and FASTQ file generation, reads were aligned against the GRCh37 reference human genome with a custom banded Smith-Waterman aligner. Single-nucleotide polymorphisms and short insertions or deletions were identified using the Genome Analysis Toolkit (Broad Institute, Cambridge, MA; *https://gatk.broadinstitute.org*). Variants were further processed by using a custom Snakemake pipeline.^28^ SnpSift version 4.3t (*https://pcingola.github.io/SnpEff*) was used for annotating variants with dbSNP version 20180423 (*https://www.ncbi.nlm.nih.gov/snp*), ClinVar (*https://www.ncbi.nlm.nih.gov/clinvar*, last accessed July 6, 2020), or COSMIC version 92 (*https://cancer.sanger.ac.uk/cosmic*) coding mutations. In addition, ENSEMBL VEP (*https://www.ensembl.org/info/docs/tools/vep/index.html*, last accessed June 26, 2020; annotation data set downloaded at the same time) was used for annotating variant consequences or impact, and allele frequency data from the 1000 Genomes, gnomAD, or ESP projects.

For reliable detection of high-confidence mutations, variants were filtered based on several criteria: for each sample, variants were excluded if coverage was <100 reads, <20 reads supported the variant allele, or the variant allele frequency (VAF) was <5%. Synonymous variants and known single-nucleotide polymorphisms were excluded (based on an overall population allele frequency of >1% according to the gnomAD database).

### Digital Droplet PCR

Screening and quantitative assessment of the *FLT3* p.D835Y mutation were performed by digital droplet PCR using a mutation-specific assay for *FLT3* p.D835Y (assay ID: dHsaMDV2010047; Bio-Rad Laboratories, Hercules, CA). Reactions were performed according to the manufacturer’s recommendations. Droplets were generated by using the QX200 Automated Droplet Generator (Bio-Rad Laboratories) followed by fluorescent signal detection with the QX200 Droplet Reader system (Bio-Rad Laboratories). Results were evaluated and quantified by using QuantaSoft software version 1.7 (Bio-Rad Laboratories). Allelic burden of the mutation was defined as fractional abundance calculated from the ratio of the number of mutant DNA molecules (a) and the total number of mutant (a) plus wild-type (b) DNA molecules detected: fractional abundance = a/(a + b). Sensitivity of the digital droplet PCR analysis was assessed for each sample; the lower limit of the quantitative range could ubiquitously be determined as 0.01%.

### Statistical Methods

Event-free survival (EFS) and overall survival (OS) were estimated by using the Kaplan–Meier method and compared statistically by using the log-rank test. Complete remission was defined as <5% blasts in the bone marrow, no evidence of leukemia at any other site, and evidence of regeneration of normal hematopoietic cells. OS was calculated from the date of diagnosis to exit or last follow-up. EFS was calculated from the date of diagnosis to the first event (induction failure, relapse, or death) or to the date of the last follow-up (death from early toxicity was excluded). Patients who failed to achieve complete remission at day 60 were considered as failures.

Comparisons between patient subgroups were performed by using the *U*-test for continuous variables and by the χ^2^ or Fisher exact test for categorical variables. All statistical tests were performed by using GraphPad Prism software version 9.2.0 (GraphPad Software, La Jolla, CA).

## Results

### Cytogenetic Characteristics of Patients at Diagnosis

**Figure 1:**
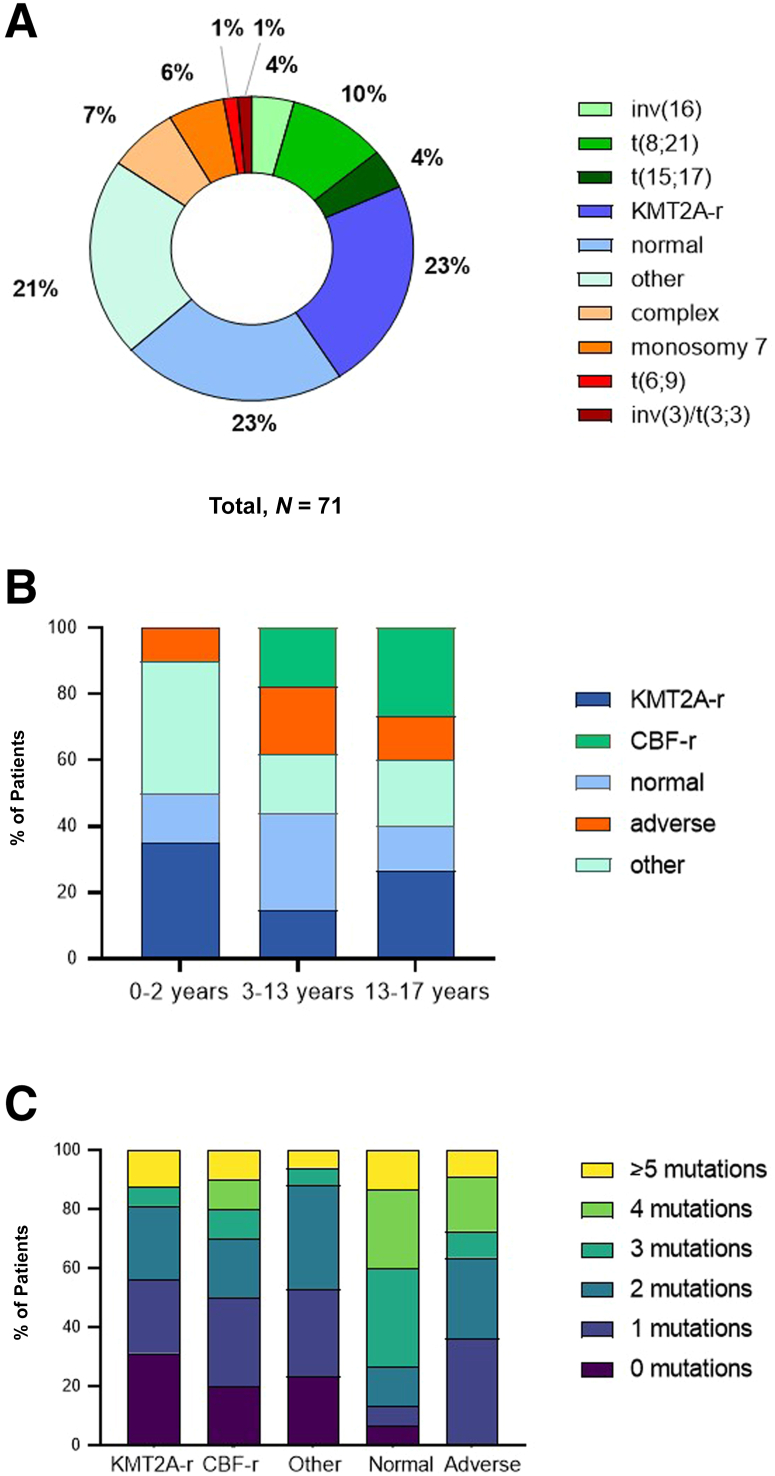
**A:** Pie chart showing the frequency of the major cytogenetic subgroups in pediatric acute myeloid leukemia. Favorable risk cytogenetic groups are shown in green, the poor-risk groups in orange, and the intermediate-risk group subgroups in blue. **B:** Distribution of the cytogenetic subgroups according to age groups. Cytogenetics appeared different according to age groups, with younger children harboring KMT2A-rearragement and “other” aberrations more frequently, whereas the ratio of core-binding factor rearrangement (CBF-r) increased with age. **C:** Number of mutations detected at diagnosis according to cytogenetic subgroup. Highest mutation rate was detected in normal karyotype acute myeloid leukemia and karyotypes associated with adverse prognosis.

Cytogenetic results were available in 71 cases, with normal karyotype detected in 21.3% (*n* = 16) of the patients (Supplemental Table S1). *KMT2A (MLL)*-rearrangements were the most frequently [*n* = 16 (21.3%)] observed cytogenetic aberrations, followed by CBF-rearrangements [*n* = 10 (13.3%)]: t(8;21) translocations (*n* = 7) and inv(16)/t(16;16) alterations (*n* = 3). Eleven patients had karyotype associated with adverse prognosis [complex karyotype, monosomy 7, t(6;9) or inv(3)]. Overall, 20.0% of the patients (*n* = 15) were characterized by “other” aberrations (ie, cytogenetic abnormalities not classified in the aforementioned subgroups) (Figure 1A). The cytogenetic categories displayed an age-related distribution, with younger children harboring *KMT2A*-rearragement and “other” aberrations more frequently, whereas the abundance of CBF-rearrangements and adverse karyotype increased with age (Figure 1B).

### Genomic Profiling Using Targeted NGS

**Figure 2:**
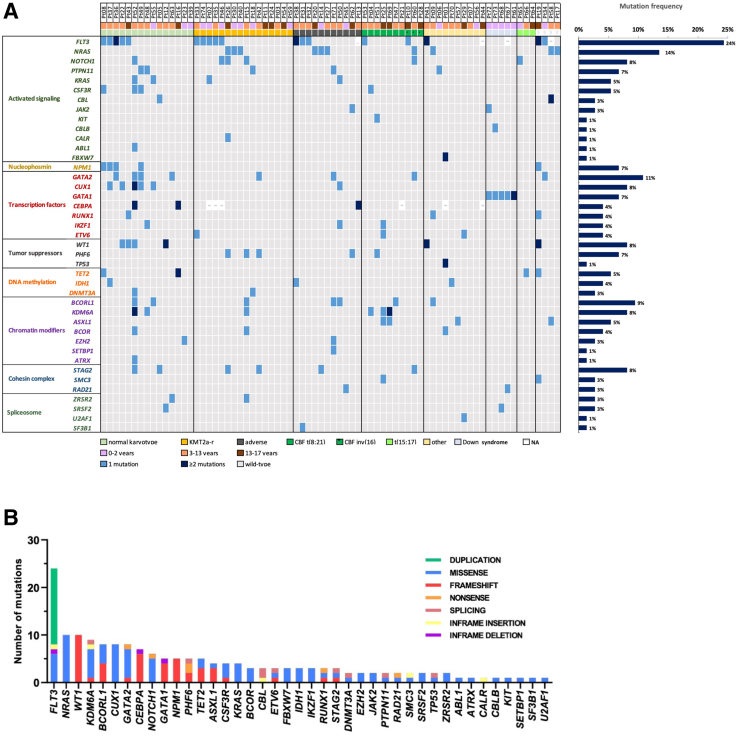
**A:** Heat map displaying the somatic variants detected in the 54 target genes analyzed in the diagnostic samples of 74 pediatric patients with acute myeloid leukemia. Illustrated is the distribution of the somatic variants, age groups, and cytogenetic profiles as determined by karyotyping or fluorescence *in situ* hybridization, as well as the mutation frequency of the individual genes for all cases. **B:** Bar graph depicting the total number of mutations detected in individual genes ranked in order of recurrence. Mutation types are distinguished by different colors. NA, not available.

**Supplemental Figure S1:**
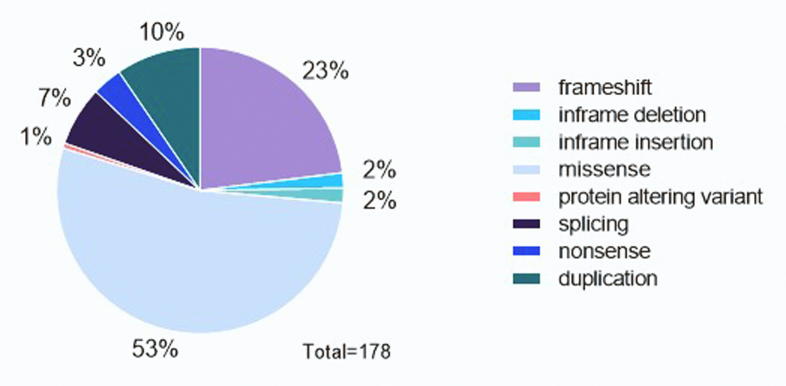
Distribution of the mutation types and frequencies in the diagnostic samples of 74 pediatric patients with acute myeloid leukemia based on next-generation sequencing analysis (*n* = 154), fragment analysis of *FLT3*-internal tandem duplication (*n* = 17) and Sanger sequencing of *CEBPA* (*n* = 7).

**Supplemental Figure S2:**
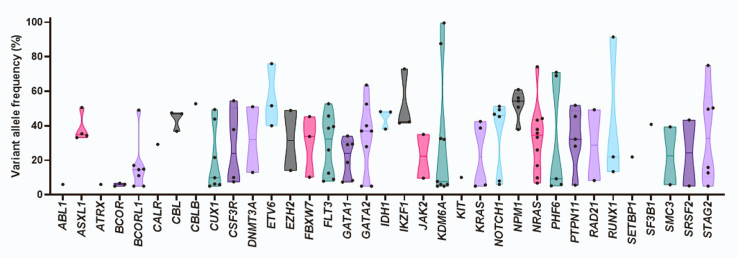
Variant allele frequencies in individual genes across the 40 genes analyzed by next-generation sequencing.

Targeted NGS with an average allelic depth of 4960× revealed, in total, 154 single nucleotide variants and short insertions/deletions in the diagnostic samples of 74 patients (Figure 2A and Supplemental Table S4). The number of mutations identified in patients at diagnosis showed uneven distribution across specific cytogenetic subgroups, although these differences were not statistically significant (Figure 1C). The median number of mutations per patient was 2.0 (range, 0 to 18), with the highest rate of mutations in cytogenetically normal AML (CN-AML) (3.0) and the lowest in *KMT2A*-rearranged AML (1.0). In terms of mutation classes, missense and frameshift mutations were the most commonly detected, followed by duplications and splice site variants (Supplemental Figure S1 and Figure 2B). Distribution of VAFs exhibited heterogeneity, with most genes being affected by both subclonal and clonal alterations. Mutations in *ASXL1*, *CBL*, *ETV6*, *IDH1*, and *NPM1* emerged with a VAF of >30% in all cases (Supplemental Figure S2). Overall, 83.8% (62 of 74) of patients carried at least one mutation in genes analyzed by NGS. Considering all genetic alterations detected by different modalities including cytogenetics, aberrations were identified in 98.6% (73 of 74) of patients (Figure 2A).

**Figure 3:**
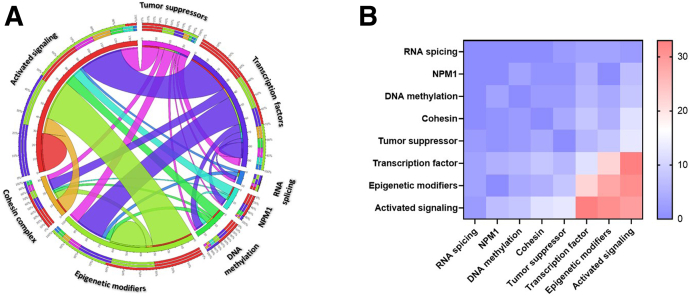
**A:** Circos plot diagram illustrating the pairwise co-occurrence of molecular aberrations based on the functional groups. **B:** Mutations associated with activated signaling commonly emerge together and with mutations of genes encoding transcription factors and epigenetic modifiers. Mutations of tumor suppressor genes occur concomitantly with mutations of other functional groups.

**Supplemental Figure S3:**
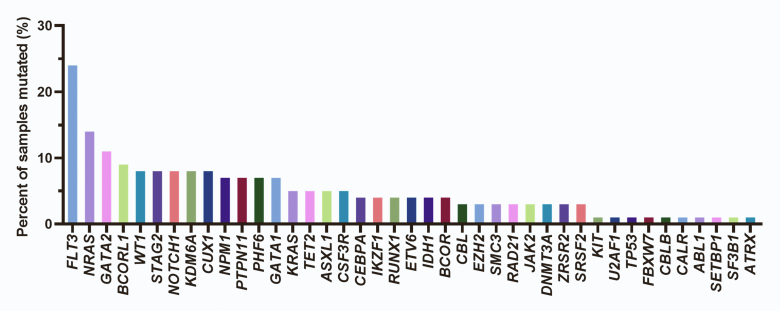
Bar plot showing mutations ranked by frequency of mutations in patients.

The most common class of mutations involved genes controlling kinase signaling [36.7% (*n* = 65)] and encoding transcription factors [20.9% (*n* = 37)], followed by chromatin modifiers [15.3% (*n* = 27)], tumor suppressors [9.6% (*n* = 17)], DNA methylation [5.6% (*n* = 10)], cohesion genes [5.6% (*n* = 10)], and RNA splicing [3.4% (*n* = 6)] (Figure 2A and Supplemental Figure S3). Figure 3A depicts the pairwise co-occurrence of the mutations in the different functional subgroups. Mutations associated with activated signaling commonly emerged together and with mutations of genes encoding transcription factors and epigenetic modifiers, whereas *NPM1* and epigenetic modifier mutations were mutually exclusive (Figure 3B). Mutations of kinase signaling genes were found in 60.8% of patients spread across all subtypes.

Of the 54 genes examined, 40 genes were altered in our cohort, with 17 genes recurrently mutated in >5% of patients. *FLT3* [24% (18 of 74)], *NRAS* [14% (10 of 74)], and *GATA2* [11% (8 of 74)] represented the most frequently mutated genes (Figure 2A). *FLT3*-ITDs were detected in 14.9% (11 of 70) of the diagnostic patient samples, including a total of 17 *FLT3*-ITD mutations with a median allelic ratio of 0.09 (range, 0.02 to 4.91), whereas length of ITD varied between 6 and 96 bp (Supplemental Table S5). The majority [63.6% (7 of 11)] of *FLT3*-ITD–positive cases carried a single mutation, whereas 18.2% (2 of 11) of patients harbored two mutations and 18.2% (2 of 11) harbored three mutations. As expected, *FLT3*-ITD mutations were predominantly present in patients with CN-AML [27% (4 of 15)] and CBF-AML [20% (2 of 10)].

Following *FLT3*-ITD mutations, alterations of the tyrosine kinase domain (TKD) of *FLT3* was the most frequently observed, as *FLT3*-TKD mutations were present in 8.1% (6 of 74) of patients: mutations occurred in codons 835 (*n* = 4), 836 (*n* = 1), and 841 (*n* = 1). Of the six patients with an *FLT3*-TKD mutation, four patients had *KMT2A*-rearrangement; conversely, 31.0% (5 of 16) of patients with *KMT2A*-rearrangements harbored *FLT3* mutation, and interestingly, no *FLT3*-ITD mutation was found in this subgroup (only *FLT3*-TKD). In addition, a single patient harbored a point mutation at the juxtamembrane domain of *FLT3*.

RAS pathway mutations (*NRAS*, *KRAS*, *PTPN11*, and *CBL*) were present in 27.0% (20 of 74) of patients and mainly occurred in *KMT2A*-rearranged cases and CN-AML. Most *NRAS* mutations (7 of 10) were found at codon 61, whereas mutations at codon 12 and 13 were detected in 2 cases and 1 case, respectively. The median VAF of *NRAS* mutations was 35% (7% to 74%).

*NPM1* mutations were detected in 6.8% (5 of 74) of patients. Four of the five mutations had been previously reported: three patients had type A mutation (c.863_864dupTCTG), one patient had type J mutation (c.863_864insCCGG), and one patient presented with a novel *NPM1* mutation (c.869_873delinsCCCTTTCCC). Four of the five patients with *NPM1* mutation harbored concomitant *FLT3* mutations. *NPM1* mutation was associated with normal karyotype (*P* = 0.0015), in line with previous publications.^13^, ^29^

*KDM6A* mutations were present in 8.1% of patients (6 of 74) at diagnosis with close association with CBF-rearrangements, as 30% (3 of 10) of patients with CBF-AML harbored *KDM6A* mutations (*P* = 0.0343). Notably, *KDM6A* mutations were restricted to patients with t(8;21) AML, whereas they were absent in inv(16)/t(16;16). Regarding CBF-rearranged AML, no enrichment of *KIT* or *RAS* mutations was found in this subgroup of our cohort.

In addition to genes with known mutational hotspots (*NRAS*, *FLT3*, *IDH1*, *ASXL1*, and *SRSF2*), recurrent variants were detected in *CUX1* and *BCORL1* genes. *CUX1* c.1573C>G p.Leu525Val and c.1613A>G p.Asp538Gly variants were detected in 2 and 2 patients, respectively, with VAFs ranging from 6% to 44%. Overall, *CUX1* mutations were detected in 8% (6 of 74) of patients, predominantly associated with CN-AML (5 of 6; *P* = 0.0013). Nine percent (7 of 74) of patients carried *BCORL1* mutations, including a recurrent frameshift mutation (c.2541del p.Ser848ValfsTer5) identified in three patients, not previously described.

The entire coding region of the *CEBPA* gene was screened in 68 patients. *CEBPA* mutations were detected in three patients (4.4%), all of whom harbored both N- and C-terminal type of mutations. All *CEBPA* mutations are summarized in Supplemental Table S6. Two novel frameshift mutations were identified (c.950_953delinsACCTT p.Leu317HisfsTer4; c.691_701del p.Val232AlafsTer85) in our cohort. Of the three children with *CEBPA* mutations, two had *de novo* AML with normal karyotype, and one patient presented with therapy-associated AML with complex karyotype.

### Comparative Analysis of Mutational Profiles at Diagnosis and Relapse

**Figure 4:**
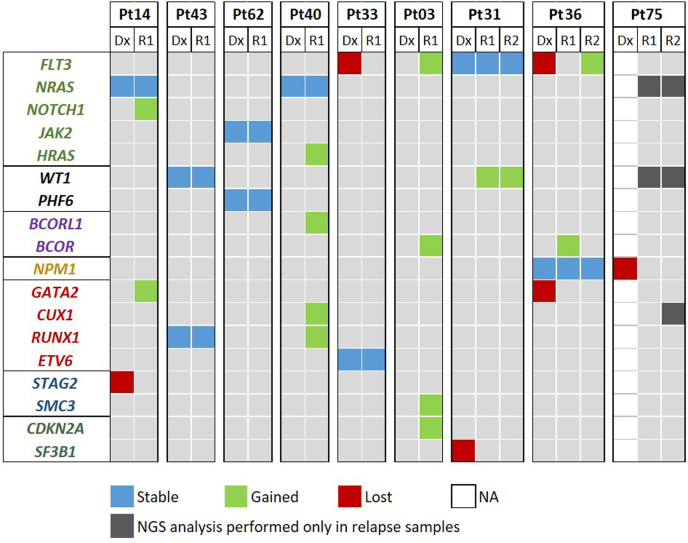
Heat map displaying the mutational status of nine patients at the time of diagnosis and relapse. Stable mutations (ie, present at diagnosis and relapse) and unstable mutations (ie, present either only at diagnosis or at relapse) are shown with different colors [DNA was not available from Patient 75 to perform next-generation sequencing (NGS) analysis at diagnosis, although the *NPM1* mutational status was known]. NA, not available; Pt, patient.

To uncover progression-related changes in mutational profile, targeted NGS analysis was performed on matched diagnosis–relapse samples of eight patients with pediatric AML. Overall, nine first-relapse samples and three second-relapse samples were analyzed (in a single case, no diagnostic DNA sample was available). The relapse samples carried a slightly higher number of mutations compared with the diagnostic samples, with an average of 2.5 mutations (range, 1 to 6) per sample in the relapsed cohort (versus 2.0 at diagnosis). Overall, 61.5% (8 of 13) of initially detected mutations persisted at relapse, and 38.5% (5 of 13) of mutations were detected only in the diagnostic sample; 65.4% of mutations (17 of 26 relapse mutation) emerged during disease progression. Mutations that persisted from diagnosis to relapse had a higher VAF at diagnosis compared with those that were eliminated at relapse (median VAF at diagnosis, 30.7% versus 10.9%). However, due to the relatively limited number of analyzed samples, this did not reach statistical significance. At relapse, mutations were identified in 16 genes, with *WT1* [42% (5 of 12)], *FLT3* [42% (5 of 12)], *NRAS* [33% (4 of 12)], and *NPM1* [25% (3 of 12)] representing the top four affected genes (Figure 4). Comparison of the function of the affected genes at diagnosis and relapse revealed enrichment of mutations of genes affecting tumor suppression (16.2% versus 44.4%) and transcription factors (35.1% versus 55.6%) at relapse (Figure 4).

**Figure 5:**
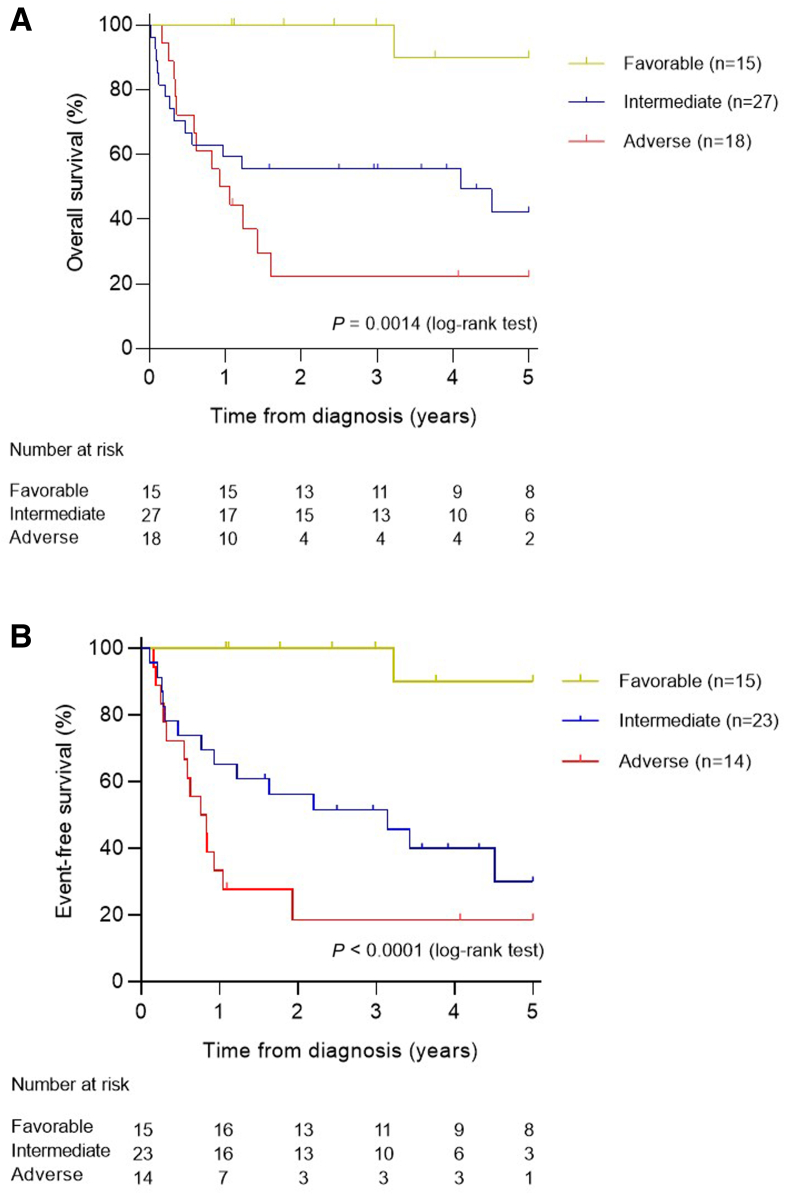
Five-year overall survival (**A**) and event-free survival (**B**) rates according to favorable (**yellow**), intermediate (**blue**), and adverse (**red**) risk groups based on cytogenetic alterations and mutational status of *NPM1*, *CEBPA*, *FLT3*-internal tandem duplication, and *WT1*. Log-rank *P* value is indicated.

**Supplemental Figure S4:**
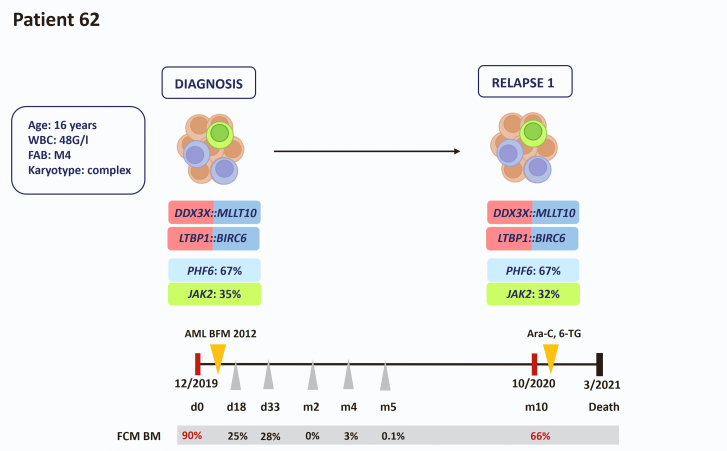
Detailed illustration of the clinical and genetic events from diagnosis to relapse. Comparison of matching mutation profiles between clones dominating at diagnosis and relapse show identical mutational profiles. Targeted RNA-sequencing show *DDX3X::MLLT10* and *LTBP1::BIRC6* fusion genes in Patient 62 (confirmed with Sanger sequencing).

**Supplemental Figure S5:**
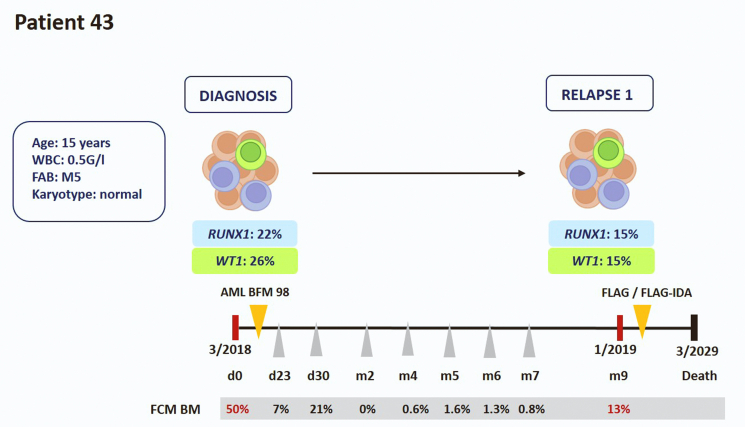
Detailed illustration of the clinical and genetic events from diagnosis to relapse. Comparison of matching mutation profiles between clones dominating at diagnosis and relapse revealed completely identical mutational profiles in Patient 43.

**Supplemental Figure S6:**
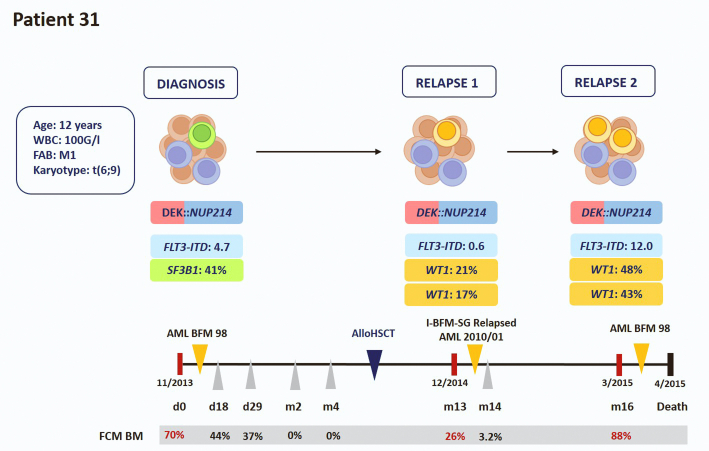
Detailed illustration of the clinical and genetic events from diagnosis to relapse. Comparison of matching mutation profiles between clones dominating at diagnosis and relapse revealed branching evolution with acquisition of additional mutations in Patient 31.

**Supplemental Figure S7:**
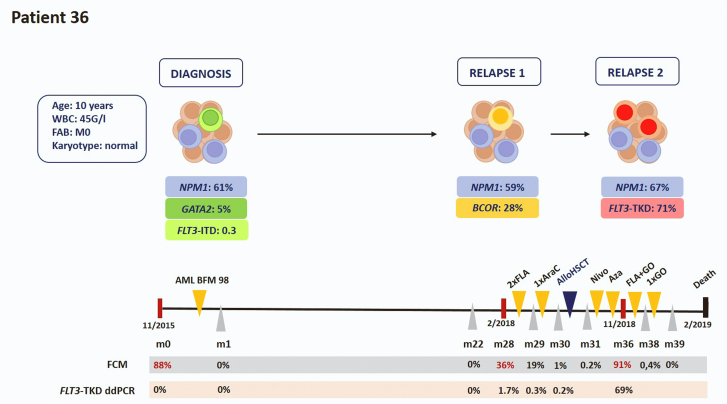
Detailed illustration of the clinical and genetic events from diagnosis to relapse. Comparison of matching mutation profiles between clones dominating at diagnosis and relapse revealed branching evolution with acquisition of additional mutations in Patient 36.

**Supplemental Figure S8:**
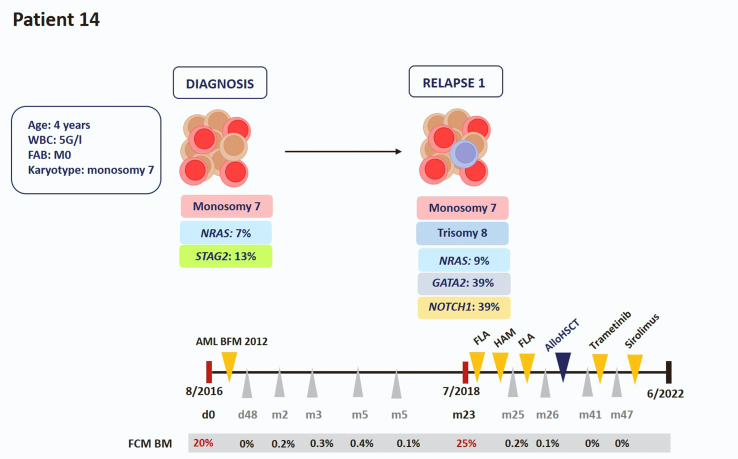
Detailed illustration of the clinical and genetic events from diagnosis to relapse. Comparison of matching mutation profiles between clones dominating at diagnosis and relapse revealed branching evolution with acquisition of additional mutations in Patient 14.

**Supplemental Figure S9:**
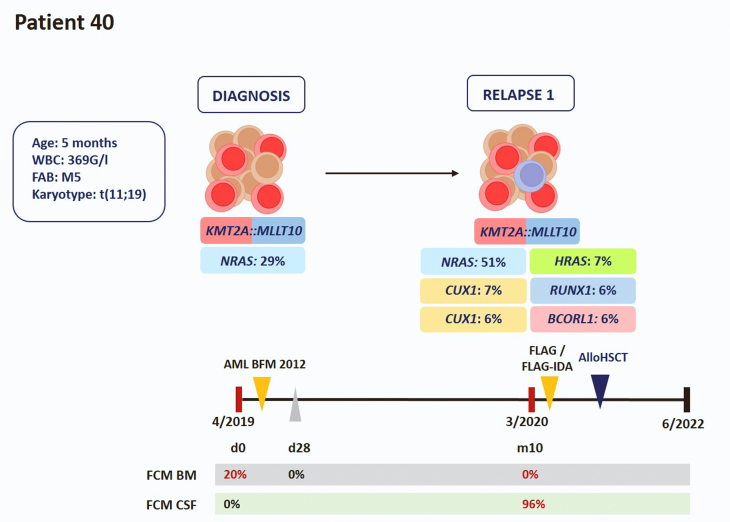
Detailed illustration of the clinical and genetic events from diagnosis to relapse. Comparison of matching mutation profiles between clones dominating at diagnosis and relapse revealed branching evolution with acquisition of additional mutations in Patient 40.

**Supplemental Figure S10:**
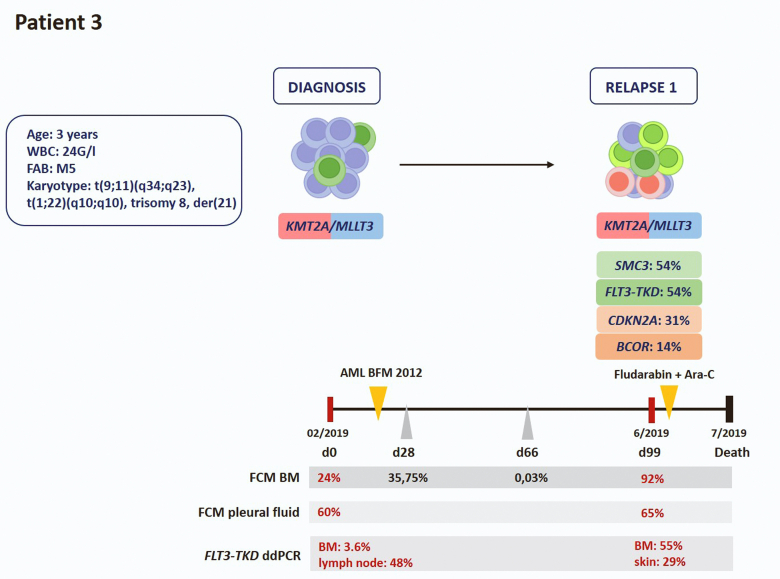
Detailed illustration of the clinical and genetic events from diagnosis to relapse. Comparison of matching mutation profiles between clones dominating at diagnosis and relapse revealed branching evolution with acquisition of additional mutations in Patient 3.

**Supplemental Figure S11:**
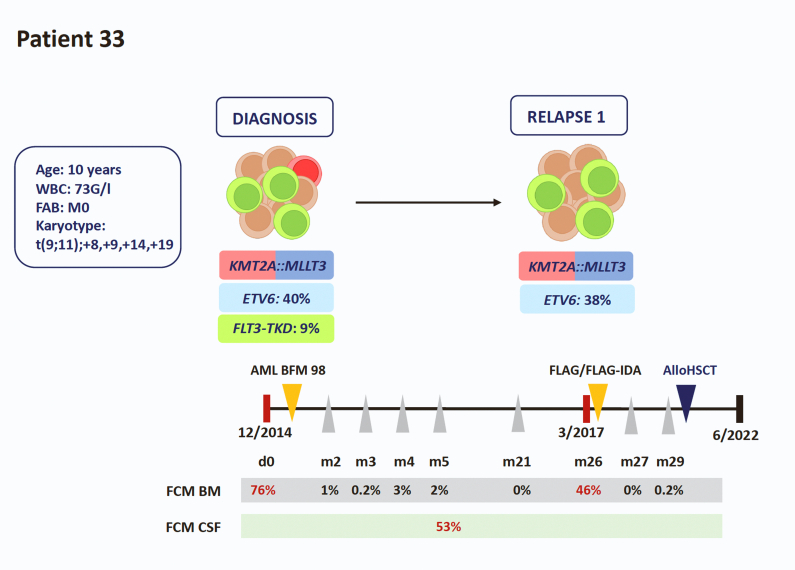
Detailed illustration of the clinical and genetic events from diagnosis to relapse. Comparison of matching mutation profiles between clones dominating at diagnosis and relapse revealed eradication of the *FLT3*-tyrosine kinase domain (TKD) mutated subclone by chemotherapy in Patient 33.

**Supplemental Figure S12:**
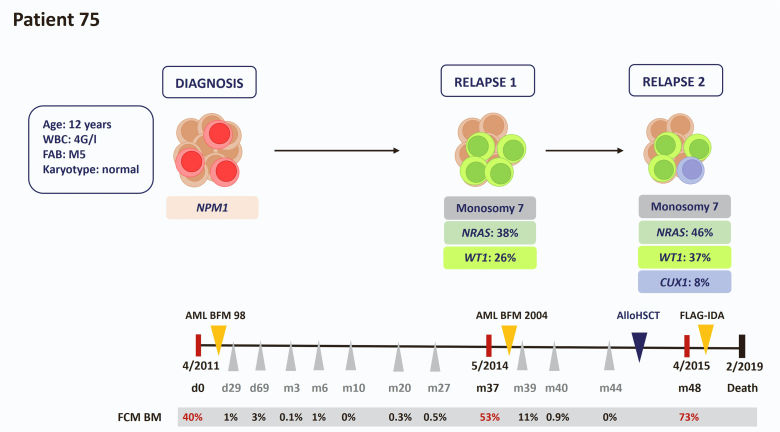
Detailed illustration of the clinical and genetic events from diagnosis to relapse. Comparison of matching mutation profiles between clones dominating at diagnosis and relapse revealed emergence of an entirely different leukemic clone in Patient 75.

Comparison of matching mutation profiles between clones dominating at diagnosis and relapse revealed three different patterns: (1) completely identical mutational profiles were observed in two patients (Patient 43 and Patient 62) at diagnosis and relapse (Supplemental Figures S4 and S5); (2) in five patients, the founding clone in the primary tumor gained additional mutations and evolved into the relapsed clone (Supplemental Figures S6–S10); and (3) in a single patient, the mutation profile slightly changed from diagnosis to relapse, as the initially detected *FLT3*-TKD mutated subclone was eradicated by the chemotherapy (Supplemental Figure S11). Patient 75 (Supplemental Figure S12) relapsed >3 years after the initial diagnosis and presented with an entirely different leukemic clone, not only in terms of the mutation profile but also the immunophenotype and the karyotype. At diagnosis, this patient presented with CN-AML with *NPM1* mutation (no DNA sample was available for targeted NGS), while at first relapse the *NPM1* mutation was eradicated and, in the meantime, monosomy 7, *NRAS*, and *WT1* mutations emerged, raising the possibility that instead of a clonally related relapse, the patient had developed therapy-related AML. After allogeneic hematopoietic stem cell transplantation, the patient experienced a second relapse from the clone detected at the first relapse carrying monosomy 7 with a newly acquired subclonal *CUX1* mutation (the presence of *NRAS*, *WT1*, and *CUX1* variants in the diagnostic sample could not be tested due to lack of DNA sample for targeted NGS).

With the exception of two patients (Patient 40 and Patient 3) who relapsed very early and showed active clonal evolution, time from diagnosis until the first relapse suggested a prolonged time requirement of clonal evolution from a founding clone compared with the quick return of an identical clone at the time of relapse (median, 24.5 versus 9.5 months) (Supplemental Figures S9 and S10).

To gain further insights into the clonal dynamics, Patient 36, who had samples available from multiple disease time points, was studied. The patient initially presented with *NPM1*, *FLT3*-ITD, and *GATA2* mutations at diagnosis; 2 years later, she eventually relapsed with a clone carrying the original *NPM1* mutation and a newly acquired *BCOR* mutation. Shortly after achieving complete remission, a second relapse evolved with the same *NPM1* mutation accompanied by a novel *FLT3* D835Y mutation. Interestingly, the *FLT3* D835Y mutation was detectable by digital droplet PCR with a low VAF (1.67%) as early as the time of the first relapse, and the mutation was still present when the patient achieved second complete remission with measurable residual disease positivity after two courses of salvage chemotherapy (VAF, 0.19%) (Supplemental Figure S3D).

### Clinical Outcome Based on Molecular Abnormalities

**Supplemental Figure S13:**
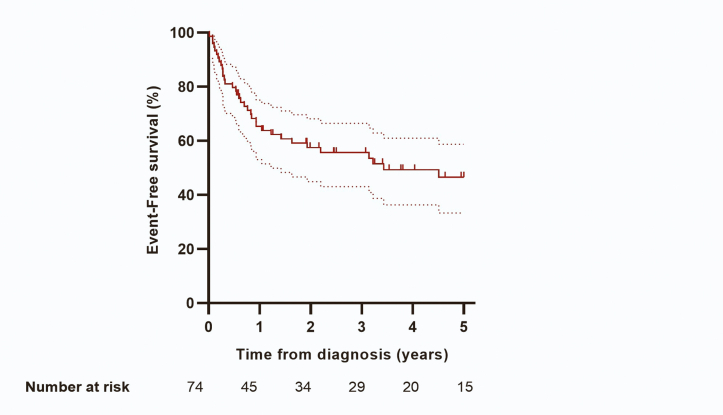
Event-free survival rates of the total cohort. The **dotted lines** represent 95% confidence intervals.

**Supplemental Figure S14:**
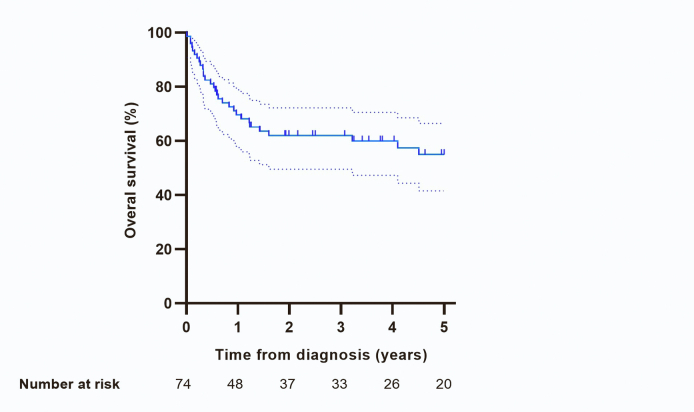
Overall survival rates of the total cohort. The **dotted lines** represent 95% confidence intervals.

At 5 years, the EFS and OS for the whole cohort proved to be 50.0% and 56.2% (Supplemental Figures S13 and S14), respectively, with a median follow-up time of 23.8 months (range, 0.2 to 205 months). Risk classification based on cytogenetics and mutational status of *NPM1*, *CEBPA*, *FLT3*-ITD, and *WT1* showed that patients in the favorable-, intermediate-, and adverse-risk categories had significantly different 5-year EFS and OS [EFS, 90% versus 30% versus 18% (*P* < 0.0001); OS, 90% versus 42% versus 22% (*P* = 0.0014)] (Figure 5). Multiparametric flow cytometry data were available from 57 patients (Supplemental Table S7). Overall, 91.8% (56 of 61) of patients achieved complete remission after two courses of intensive chemotherapy, whereas three patients experienced fatal complications during the induction therapy, and two patients were nonresponders. Due to the small number of patients, assessment of the prognostic significance of individual mutations was limited; only mutations in tumor suppressor genes (*TP53*, *PHF6*, and *WT1*) were significantly associated with induction failures (Fisher exact test, *P* = 0.0034) (Supplemental Table S8).

The prognostic significance of early response to treatment was studied at day 28. AML BFM protocols define >20% blasts on day 28 as poor response to treatment. Using this cutoff value, both the 5-year EFS and OS were significantly different between good and poor responders (57.3% versus 0%, *P* < 0.0001; 64.2% versus 28.6%, *P* = 0.0414).

Stratification based on AML-BFM protocols was as follows: patients with *RUNX1::RUNX1T1*, *CBFB::MYH11*, *PML::RARA*, normal karyotype with *NPM1*, or biallelic *CEBPA* mutations were categorized in the favorable subgroup. Patients with complex karyotype (ie, >3 aberrations), monosomy 7, t(6;9), inv(3), t(6;11), t(10;11) or *FLT3*-ITD with *WT1* mutation belonged to the adverse risk group, with all remaining patients categorized as intermediate risk. The 5-year OS was 90.0% for patients with favorable prognosis, 42.3% for patients classified in the intermediate-risk group, and 22.2% among patients with unfavorable prognosis (*P* = 0.0014); the EFS values in these risk groups proved to be 90.0%, 30.0%, and 18.5%, respectively (*P* < 0.0001).

## Discussion

Although the treatment efficacy of pediatric AML has improved over the past decades, primarily owing to increasingly intensified regimens, refined allograft indications, and supportive care, the clinical outcomes have plateaued, with 70% of patients achieving 5-year survival.^2^^,^^30^^,^^31^ Although the genomic background of AML has been widely studied, pediatric AML is less characterized, as only a limited number of studies have focused on investigating the molecular landscape of children with AML at diagnosis.^13^, ^14^, ^15^, ^16^^,^^19^, ^20^, ^21^ Genomic profiling of pediatric AML revealed low tumor mutational burden similar to adult patients, with some shared recurrently mutated genes. However, the spectrum of genetic alterations in pediatric and adult patients with AML is remarkably different, suggesting that pathogenesis of pediatric AML differs from that of the adult AML.^13^^,^^32^ Consequently, not all findings related to adult patients with AML are applicable to children, justifying the need to further investigate the mutation profiles of pediatric patients with AML.

Pediatric patients with AML are known to harbor clonal chromosomal abnormalities more frequently compared with adult AML cases, and many of these cytogenetic alterations have prognostic significance.^12^^,^^33^, ^34^, ^35^ In the current study, distribution of patients in the various cytogenetic groups was consistent with previous reports, as abnormal karyotype was detected in 77.5% (55 of 71) of pediatric patients with AML, and *KMT2A*-rearrangement and normal karyotype were the two most commonly observed genotypes. The prognostic significance of individual chromosomal abnormalities could not be evaluated due to the limited number of patients; however, risk classification based on cytogenetics clearly discriminated favorable-, intermediate-, and adverse-risk groups in our patient cohort.

The panel-based targeted NGS approach enabled us to identify single nucleotide variants and insertions/deletions in 83.8% of patients, and by combining the results with cytogenetics, at least one molecular aberration was detected in 98.6% of patients. Despite investigating only a limited number of genes, each patient had a unique genomic profile due to the unique co-occurrence of mutations. As expected, the number of variants per case was relatively low, reflecting the low tumor mutational burden previously observed in pediatric AML.^13^^,^^14^

**Supplemental Figure S15:**
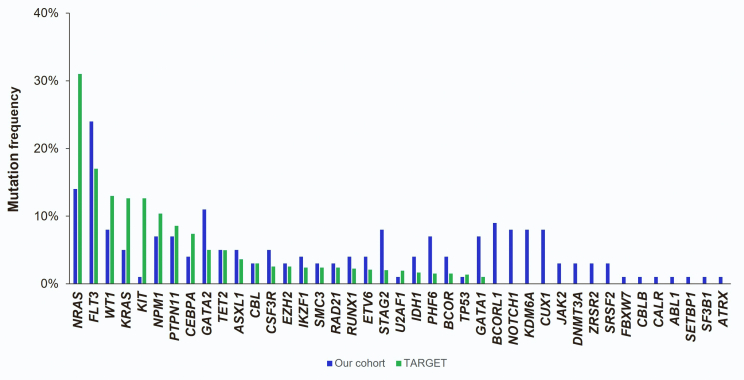
Differences in mutation frequencies of key myeloid genes in our cohort compared with TARGET (Therapeutically Applicable Research to Generate Effective Treatments) AML results. It should be noted that some of the differences in individual gene mutation frequencies between the two cohorts may be attributed to the different sequencing technologies (deep targeted next-generation sequencing versus whole exome sequencing) used in these studies as well as to the limited size (*n* = 75) of our patient cohort. Notably, our cohort included five cases of Down syndrome acute megakaryocytic leukemia and three cases of acute promyelocytic leukemia, whereas these entities were absent from the TARGET AML cohort.

*KMT2A*-rearranged cases tended to have fewer mutations than other subtypes of AML, in line with previous publications.^19^^,^^20^*KMT2A*-rearrangements are associated with early-onset AML; however, in our cohort, more than one-half of patients with *KMT2A*-rearrangement were older than 2 years. An increased number of mutations were detected in patients with normal karyotype and karyotypes associated with adverse prognosis. In our cohort, the most common class of mutations involved genes that control kinase signaling and encode transcription factors, whereas mutations in epigenetic components or spliceosome complexes commonly occurring in adult patients with AML were present less common, in line with the literature.^9^^,^^17^^,^^36^ Comparison of the mutation landscape revealed considerable differences in the mutational frequencies in some of the affected genes (Supplemental Figure S15).

RAS pathway mutations (*NRAS*, *KRAS*, *PTPN11*, and *CBL*) occurred less commonly in our cohort, as 27% of patients carried a RAS pathway mutation at diagnosis compared with 40% to 50% frequencies reported in previous publications.^13^^,^^18^^,^^20^^,^^32^ *KIT* mutations were less frequently observed, with only a single CBF-AML patient carrying a *KIT* mutation, while *KIT* mutations are detected at a significantly higher ratio (20% to 40%) in pediatric patients with CBF-AML in the literature. However, this may also be attributable to the low case number in this subgroup.^37^, ^38^, ^39^

In our cohort, *BCORL1*, *CUX1*, *KDM6A*, *PHF6*, and *STAG2* mutations were detected at higher frequency than in any other previous publications predominantly using whole-genome sequencing and whole-exome sequencing.^13^^,^^20^^,^^32^^,^^40^ Mutations in *BCOR* and its homolog, *BCORL1*, were first described in adult AML presenting in 5% to 10% of patients (mostly detected in secondary- and therapy-related AML) associated with inferior outcome.^41^, ^42^, ^43^ In children with AML, *BCOR*/*BCORL1* mutations were present in only 2.9% to 3.4% of patients.^13^^,^^19^^,^^20^^,^^40^ Assessment of clonality using VAF values suggested that the vast majority of *BCOR*/*BCORL1* mutations were subclonal, which might explain the significantly higher frequency of *BCOR*/*BCORL1* mutations in our cohort.

Interestingly, *CUX1* mutations detected in 8% of our cohort were not identified in the previous pediatric AML studies based on whole-genome/whole-exome sequencing and were detected in only a single patient with pediatric AML by Tarlock et al using deep-sequencing.^13^^,^^16^^,^^19^^,^^32^ *CUX1* is a transcription factor regulating cell cycle progression and apoptosis and acts as a haploinsufficient tumor suppressor frequently impaired in myeloid neoplasms, mainly through loss of chromosome 7.^9^^,^^44^^,^^45^ *CUX1* mutations are associated with inferior prognosis in myeloid malignancies, similar to the adverse outcome of –7/(del7q) myeloid malignancies.^44^^,^^46^ The higher frequency of *CUX1* mutations detected in our cohort may be explained by the use of deep sequencing that enabled us to identify *CUX1* mutations even at a subclonal level. *KDM6A* was recurrently altered in our cohort (8.1%) and associated with *RUNX1*::*RUNX1T1*-rearrangement, in contrast with previous publications in which *KDM6A* mutations were only described in 0.5% to 3% of pediatric patients with AML, and enrichment of *KDM6A* mutation has not been previously described in children with CBF-AML.^16^^,^^21^^,^^32^ However, studies on adult CBF-AML showed that mutations of chromatin modifiers (including *KDM6A* as well) were observed almost exclusively in AML with *RUNX1*::*RUNX1T1* fusion.^47^^,^^48^

Another X-linked tumor suppressor gene recurrently mutated in our cohort was *PHF6*. *PHF6* mutations are rare events in pediatric AML, as only 1.4% to 3.6% of patients harbor *PHF6* mutations with an associated poor outcome.^13^^,^^20^^,^^49^ Recently, Stratmann et al^50^ showed that mutational frequency of *PHF6* was substantially higher in relapsed or primary resistant patients than previously reported in diagnosis-only cohorts. This finding supports the association of *PHF6* mutations with poor outcome also reported in our study and by Marceau-Renaut et al.^20^

Risk stratification of pediatric AML is mostly based on cytogenetics; however, some recurrent mutations such as *NPM1*, *CEBPA*, and *FLT3-ITD* with *WT1* are also included in most risk classifications.^41^^,^^51^ Recently, several publications suggested optimization of risk stratifications of AML by incorporating additional genetic lesions.^52^, ^53^, ^54^, ^55^ The newly published risk classification of the European LeukemiaNet recommends mutational analysis of genes, including *BCOR*, *EZH2*, *SF3B1*, *SRSF2*, *STAG2*, *U2AF1*, and *ZRSR2* as these alterations are associated with high-risk features and adverse prognosis.^56^ Unfortunately, prognostic stratifications developed for adults are not necessarily suitable for children; these initiatives draw attention, however, to the clinical need to further investigate how newly identified molecular alterations of pediatric AML could be incorporated into the current risk classifications.

Most studies in the field of pediatric AML have focused on the prognostic significance of a single gene or a subset of genes; however, the true heterogeneity of pediatric AML could not be captured with these approaches. Only a limited number of studies evaluated the prognostic significance of mutations in childhood AML in the era of high-throughput sequencing. The favorable prognostic impact of *NPM1* and *CEBPA* mutations has been well established for many years, whereas the prognostic significance of other molecular alterations is less defined.^29^, ^57^, ^58^, ^59^ Marceau-Renaut et al^20^ showed that *PHF6* and *RUNX1* mutations are associated with poor prognosis in childhood AML, and Umeda et al^60^ showed that *UBTF* tandem duplication is a recurrent lesion in pediatric AML and is associated with poor outcomes. Due to the limited size of our cohort, the prognostic significance of individual mutations could not be comprehensively investigated. However, results from our current analysis suggest that tumor suppressor gene mutations (*PHF6*, *TP53*, and *WT1*) are associated with induction failure and a trend toward shorter EFS, although these findings will need to be confirmed in independent, larger studies.

Our study also compared the mutational landscape of eight matched diagnosis and relapse pediatric AML samples using panel-based targeted NGS. A slightly higher number of mutations was found at relapse compared with the diagnostic samples, and mutations persisting at relapse had higher VAF at diagnosis compared with those that were eliminated. Relapse-specific mutation could not be identified, although mutations in tumor suppressor genes and epigenetic modifiers occurred more frequently at relapse. In five patients, relapsed AML evolved from one of the subclones detected at the initial diagnosis and was accompanied by several additional mutations that were absent or present at a lower allele frequency in the diagnostic sample, indicating the multistep process of leukemia recurrence. In contrast, two patients relapsed early with seemingly identical clones, suggesting the incomplete eradication of leukemic cells by the initial treatment.

Targeted NGS is a robust tool for the reliable detection of disease-relevant alterations that determine distinct genetic subgroups of pediatric AML and/or are associated with disease prognosis. The comprehensive profiling of recurrent genomic aberrations achieved by examining a high number of different genes also provides valuable information at both the time of diagnosis and relapse. Our results further show the versatile applicability of targeted NGS and strongly support its future incorporation into the clinical research and diagnostic workflow of pediatric patients with AML.

In summary, we performed an integrative analysis of cytogenetic and molecular profiles of pediatric AML from a multicentric, real-world patient cohort and identified previously unappreciated alterations, potentially leading to improved molecular characterization and risk stratification of patients with pediatric AML. To the best of our knowledge, this study is the first to characterize the mutational landscape of pediatric patients with AML treated according to AML BFM protocols in Central Europe. Our results also show that childhood AML represents a distinct entity that differs from adult AML in terms of the spectrum of gene mutations.
